# The auriculo-vagal afferent pathway and its role in seizure suppression in rats

**DOI:** 10.1186/1471-2202-14-85

**Published:** 2013-08-09

**Authors:** Wei He, Xiang-Hong Jing, Bing Zhu, Xin-Long Zhu, Liang Li, Wan-Zhu Bai, Hui Ben

**Affiliations:** 1 Institute of Acupuncture and Moxibustion, China Academy of Chinese Medical Sciences, 16 Nanxiaojie, Dongzhimennei, Beijing 100700, China

## Abstract

**Background:**

The afferent projections from the auricular branch of the vagus nerve (ABVN) to the nucleus tractus solitaries (NTS) have been proposed as the anatomical basis for the increased parasympathetic tone seen in auriculo-vagal reflexes. As the afferent center of the vagus nerve, the NTS has been considered to play roles in the anticonvulsant effect of cervical vagus nerve stimulation (VNS). Here we proposed an “auriculo-vagal afferent pathway” (AVAP), by which transcutaneous auricular vagus nerve stimulation (ta-VNS) suppresses pentylenetetrazol (PTZ)-induced epileptic seizures by activating the NTS neurons in rats.

**Results:**

The afferent projections from the ABVN to the NTS were firstly observed in rats. ta-VNS increased the first grand mal latency of the epileptic seizure and decreased the seizure scores in awake rats. Furthermore, when the firing rates of the NTS neurons decreased, epileptiform activity manifested as electroencephalogram (EEG) synchronization increased with 0.37±0.12 s delay in anaesthetized rats. The change of instantaneous frequency, mean frequency of the NTS neurons was negative correlated with the amplitude of the epileptic activity in EEG traces. ta-VNS significantly suppressed epileptiform activity in EEG traces via increasing the firing rates of the neurons of the NTS. In comparison with tan-VNS, the anticonvulsant durations of VNS and ta-VNS were significantly longer (*P*<0.01). There was no significant difference between the anticonvulsant durations of VNS and ta-VNS (*P*>0.05). The anticonvulsant effect of ta-VNS was weakened by reversible cold block of the NTS.

**Conclusions:**

There existed an anatomical relationship between the ABVN and the NTS, which strongly supports the concept that ta-VNS has the potential for suppressing epileptiform activity via the AVAP in rats. ta-VNS will provide alternative treatments for neurological disorders, which can avoid the disadvantage of VNS.

## Background

The innervations of the external ear have contributions from cranial nerves and cervical nerves [1]. Among these innervations, the auricular branch of the vagus nerve (ABVN), which is often termed the Alderman's nerve or the Arnold's nerve, mainly innervates the external auditory canal and the auricular concha. Arnold's reflexes manifested as hyperactive vagal responses have been clinically observed [2,3]. Previous studies preliminarily proposed that the Arnold's reflexes might be mediated via the afferent connections from the ABVN to the nucleus tractus solitarius (NTS) [4-7].

The NTS not only plays important roles in autonomic regulations, but also receives extensive projections from other brain structures. Both the autonomic system and the central nervous system could be modified by auricular vagal stimulation via projections from the ABVN to the NTS, herein referred to as the “auriculo-vagal afferent pathway” (AVAP).

Epilepsy is a disorder of the brain characterized by an enduring predisposition to generate epileptic seizures and by the neurobiologic, cognitive, psychological, and social consequences of this condition. Left cervical vagus nerve stimulation (VNS) has been approved by the U.S. Food and Drug Administration (FDA) as an adjunctive treatment for refractory partial-onset seizures in adults and adolescents [8]. VNS attenuates seizure frequency, seizure severity, and is associated with positive changes in mood, memory and quality of life [9]. The anti-seizure effect of VNS is considered to be mediated via vagal afferent projections to the NTS, then via pathways from the NTS to other brain structures which correlate with the pathogenesis of epilepsy, with a result to electroencephalogram (EEG) desynchronization [10].

Together, these studies suggest the hypothesis that transcutaneous auricular vagus nerve stimulation (ta-VNS) suppresses epileptic seizures via the AVAP. In the present study, afferent projections of the ABVN in rats have been studied by using morphological techniques. Then behavioural and electrophysiological experiments in rats were performed to explore the roles of the AVAP in the anticonvulsant effect of ta-VNS.

## Methods

Five different animal experiments were performed in the present study, including (1) afferent projections of the ABVN in rats by using cholera toxin subunit B (CTB) tracing technique, (2) ta-VNS on t seizures in awake rats, (3) ta-VNS on electroencephalography (EEG) and the firing of the NTS neurons in anaesthetized rats, (4) ta-VNS on the field potentials (FPs) of the primary somatosensory in anaesthetized rats, and (5) the effect of reversible cooling the NTS neurons on epileptiform activity in EEG traces in anaesthetized rats. ta-VNS, transcutaneous auricular non-vagus nerve stimulation (tan-VNS), or vagus nerve stimulation (VNS) were performed in experiment (2) – (5) as needed to observe the anticonvulsant efficacy of the ta-VNS. The detailed experimental procedures are as follows.

### Animals

Totally ninety four adult male healthy rats, Sprague Dawley, weighing 250–320 g, were used. Rats feeding with normal diet were housed in a room maintained at 24±1°C and illuminated for 12 hours (07:00 to 19:00) every day. Food and water were freely available. Rats were allowed to adapt to the environment for 7 days prior to the experiment. Epileptiform activity was evoked by intraperitoneal injection of pentylenetetrazol (PTZ, 60 mg/kg; Sigma-Aldrich, St. Louis, MO, U.S.A.). For anaesthesia, 10% urethane (1.2 g/kg, via intraperitoneal route) was initiated. Additional sodium pentobarbital was administered to prolong the anaesthetic state. Animals were euthanized by urethane over-dose at the end of the study. All studies were approved by the Institutional Animal Care and Use Committee of China Academy of Chinese Medical Sciences and were in accordance with National Institutes of Health guidelines.

### Tracing study on central projections of primary afferent fibers of the ABVN

Microinjection was carried out on 9 rats. Under anaesthesia, microsyringe contained 1% CTB (List Biological Labs, Campbell, CA, USA) solution was inserted subcutaneously into the junction of cavity of the auricular concha and postero-inferior wall of the external acoustic meatus for a depth of 2–3 mm along the auricular surface, and a total of 3 μl 1% CTB was injected slowly. After injection, the needle was retained about one min and then withdrawn carefully. On the third surviving day, the rats were deeply anaesthetized with ether (Beijing Chemical Plant, Beijing, China) and transcardially perfused with 100 ml of 0.9% saline and immediately followed by 300 ml of 4% paraformaldehyde in 0.1M phosphate buffered solution (PB, pH 7.4).

The brain stem, cervical spinal cord and cervical ganglia were dissected out and stored in 30% sucrose PB at 4°C and allowed to sink. Serial sections of brain stem, cervical spinal cord and cervical ganglia were cut at a thickness of 40 μm on a freezing microtome (MICROM, HM 400 R, Walldorf, Germany) and collected in 0.1M PB. The sections were processed for immunofluorescence staining. In brief, sections were incubated in a blocking solution containing 3% normal rabbit serum and 0.3% Triton X-100 in 0.1M PB for 1 hr, then transferred to goat anti-CTB (List Biological Labs) at a dilution of 1:1000 in 0.1M PB containing 1% rabbit serum and 0.3% Triton X-100 for overnight at 4°C. On the following day, after washing three times with 0.1M PB, sections were exposed to rabbit anti-goat Alexa 488 secondary antibody (1:500; Molecular Probes, Eugene, OR, USA) for 2 hr and then washed with 0.1M PB. Slides were coverslipped using Immu-mount (Thermo Shandon, Pittsburgh, USA) to improve visualization of labeling. The anatomical structure of tissue sections was determined cytoarchitecturally based on *The Rat Brain in Stereotaxic Coordinates* (Paxinos and Watson, 1998). The tissue samples were observed and recorded with a fluorescent microscope (Y-IDP; Nikon Co., Tokyo, Japan) equipped with a digital camera (DMX1200C; Nikon, Japan). Digital images were finally processed with Adobe Photoshop CS2 (Adobe Systems, San Jose, CA, USA).

### Stimulations

**Figure 1:**
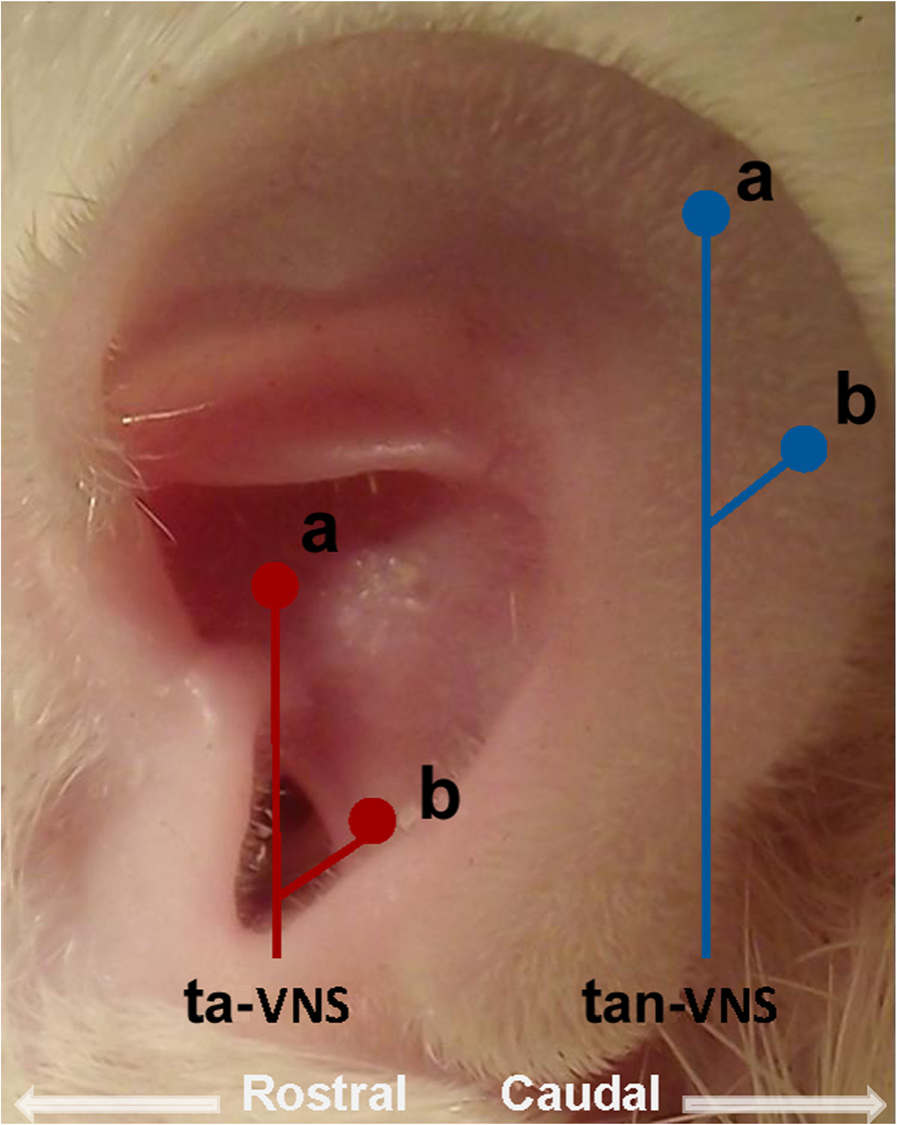
**Stimulation positions at the auricle.** Stimulations were performed at two positions of the external ear respectively. The ta-VNS was marked as two red dots. The tan-VNS was marked as two blue dots. For stimulation, bipolar sliver electrodes were attached to the skin with cathode on the position marked as “**a**”, and anode on the position marked as “**b**”.

Bipolar silver slice electrodes (diameter of 1.5 mm) being attached to the skin with adhesive tape were introduced for ta-VNS or tan-VNS. For ta-VNS, the cathode of the electrodes was placed at the junction of cavity of the auricular concha and postero-inferior wall of the external acoustic meatus, the anode was placed at the cymba of auricular concha. For tan-VNS, the electrodes were placed on the exterior margin of the auricle outside the ABVN distribution (See Figure 1). There was 3 mm distance between the two stimulation electrodes. Operative procedure of VNS was performed as described by Dorr and Debonnel [11]. For stimulations, the electrode leads were connected with stimulator (SEN-7203 Nihon Kohden, Japan). Stimulation parameters were selected as: frequency, 20 Hz; pulse width, 0.5 ms; strength, 1.0 mA.

### Observation on epileptic behaviour

Thirty awake rats were divided into three groups: control group (*n*=10), tan-VNS group (*n*=10), ta-VNS group (*n*=10). Rats in the control group were treated with intraperitoneal injection of PTZ. Rats in the tan-VNS group were firstly treated with tan-VNS for 30 min, then treated with intraperitoneal injection of PTZ. Rats in the ta-VNS group were firstly treated with ta-VNS for 30 min, then treated with intraperitoneal injection of PTZ. After intraperitoneal injection of PTZ, the latency of the first grand mal and the scores of epileptic seizures in the rats were observed within 30 min. Epileptic behaviours were scored according to the standard of Racine′s scale [12]. The observers of behavioral seizures were blinded to the treatment.

### Simultaneous recording of EEG and the extracellular discharges of the NTS neurons in anaesthetized rats

Thirty one anesthetized rats were fixed on the stereotaxic apparatus with the incisor bar placed 3.3 mm below the interaural line. After craniotomies, bipolar silver globe electrodes (diameter of 1 mm) were placed over the dura in two holes (distance to the bregma, AP: ±1.0 mm, ML: 2.0 mm) respectively for the recording of epidural EEG. EEG signals were amplified by Amplifier WPI (World Precision Instrument, Sarasota, USA) with low frequency filter at 1 Hz and high frequency filter at 100 Hz. The extracellular discharge signals of the NTS neurons (distance to the bregma, AP: -11.3 ~ −14.3 mm; ML: 0 ~ 1.3 mm, DV: 4 ~ 7 mm) were recorded by glass microelectrodes (10-20MΩ, pulled by Narishige PE-2 vertical puller from a filamented glass) which were backfilled with 2% pontamine sky blue. Firings of the NTS neurons recorded from the glass electrodes were fed through an Xcel-3 microelectrode amplifier (FHC, Bowdoin, USA) with low frequency filter at 1 Hz and high frequency filter at 15 KHz. Both signals were captured online and analyzed offline using the CED 1401-plus data acquisition system and the Spike 2 package (Cambridge Electronic Devices, Cambridge, UK). After recording, the site was histological fixed for locating recording site. Data out of the site of the NTS were deleted.

In each rat, signals at baseline were recorded for five min firstly. Then the rat was treated with intraperitoneal injection of PTZ. Signals after PTZ were recorded for five min. After PTZ, tan-VNS, ta-VNS and VNS were randomized to stimulate in the rats for 30 s respectively. There was an interval of 30 min before beginning the next stimulation.

### Microelectrode array recording of FPs in primary somatosensory in anaesthetized rats

The array with sixteen microelectrodes were implanted into the somatosensory cortices for use in recording FPs after craniotomy in 12 rats after anaesthesia. After being inserted in the correct position, the microelectrodes were cemented to skull screws by use of dental cement. FPs were collected by using Cerebus™ 5.0 Data Acquisition System at a sampling rate of 1000 Hz and a bandpass at 1–100 Hz, and were analyzed by Spike 2 package.

Firstly the rats were treated with intraperitoneal injection of PTZ. After PTZ, tan-VNS, ta-VNS were randomized to stimulate in the rats for 30 s respectively. There was an interval of 30 min before beginning the next stimulation.

### Reversible cooling the NTS neurons on the EEG changes in anaesthetized rats

Twelve anaesthetized rats were fixed on the stereotaxic apparatus. An occipital craniotomy was performed in order to gain access to the dorsal surface of the medulla oblongata. A portion of the cerebellum was aspirated so that the dorsal surface of the medulla could be clearly visualized 3 mm caudal and 3 mm rostral to the obex. The bottom of a V-shaped glass tube was gently placed on the region equivalent to the area of the NTS around the obex. In order to keep the function of the NTS neurons reversible, –4°C ethylene glycol (≥98% radiochemical purity basis, PLC, aqueous solution, Sigma) was injected through the V-shaped glass tube to cool the NTS neurons physically.

There were steps for this experiment. The rats were treated with following steps including (a) intraperitoneal injection of PTZ, (b) ta-VNS for 30 s, (c) cooling of the NTS, (d) ta-VNS for 30 s during cooling of the NTS, (e) removing cooling of the NTS, (f) ta-VNS for 30 s. In the whole experiment, the EEG signals of the rats were recorded continuously.

### Data analysis

In EEG tracings or FPs tracings, an epileptiform activity was shown as a highly synchronous and large-amplitude activity at least 3 times the amplitude of baseline. The firing rate of the NTS neurons was calculated in 30 s. Data were analysed with SPSS 10.0 program (SPSS Inc. Chicago, IL). Values are presented as mean ± SEM. Kolmogorov-Smirnof test was used to evaluate if groups fit normal distributions. Normally distributed groups were analysed by parametric tests. Comparison between two groups was analysed by Independent-Samples T test. Comparison between three groups was analysed by One-Way ANOVA followed by *LSD* or Dunnett's T3 post-hoc test. Nonnormally distributed groups were analysed by Mann–Whitney test. *P* < 0.05 was considered significant.

## Results

### Afferent projections from the auricular concha to the NTS were demonstrated in the rats

**Figure 2:**
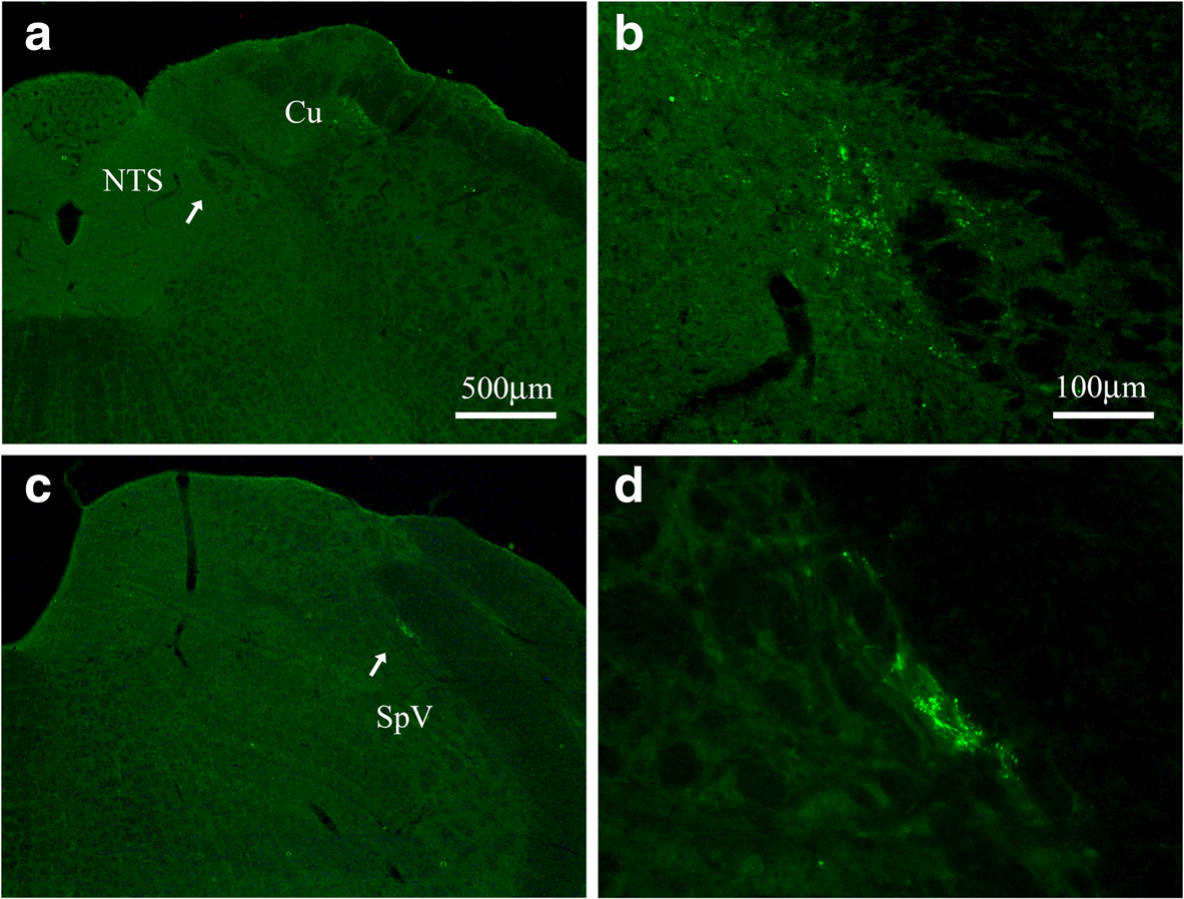
**CTB-labeled transganglionic axonal terminals at the NTS and the SpV.** Coronal sections through the medulla oblongata showing the CTB-labeled transganglionic axonal terminals at NTS **(a, b)** and SpV **(c, d)**. Higher magnified photos **(b, d)** from **a** and **c** (arrow head) showing the labeled terminals in detail respectively. Same scale bar for **a** and **c**, and for **b** and **d**. Cu, cuneate nucleus; NTS, nucleus tractus solitarius; SpV, spinal trigeminal nucleus.

Under the fluorescent microscope, CTB-labeled terminals appeared in green fluorescent granules and were located ipsilaterally on the injection side. Transganglionically labeled fibers terminated on the caudal part of lateral NTS (Figure 2a, b) and the dorsomedial edge of spinal trigeminal nucleus (SpV) (Figure 2c, d), as well as the lateral part of rostral cuneate nucleus and spinal dorsal horn of 2–3 cervical segments (not shown).

### ta-VNS pre-treatment suppressed epileptic behaviour in awake rats

**Figure 3:**
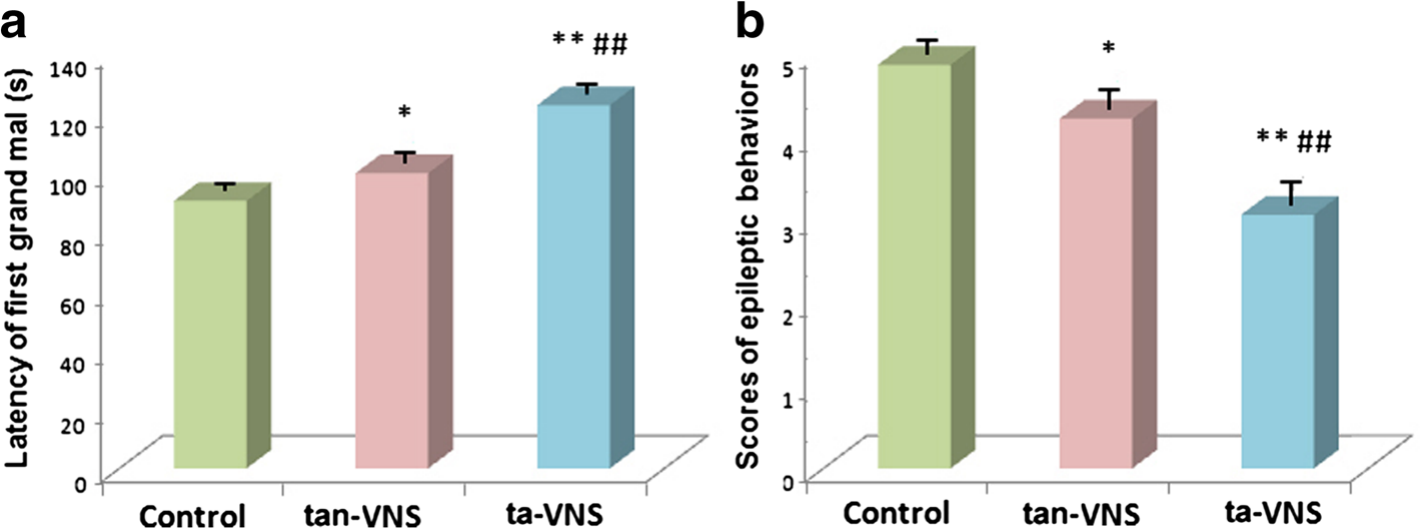
**ta-VNS suppressed epileptic behaviour in awake rats. (a)** Comparison of the latency of the first grand mal among three groups. **(b)** Comparison of the scores of epileptic behavior among three groups. *: *P* < 0.05, *vs*. the control group, **: *P* < 0.01, *vs*. the control group, ##: *P* < 0.01, *vs*. the tan-VNS group, *n*=10 per group.

In comparison with the control group, the latency of the first grand mal of the rats increased in the tan-VNS group and the ta-VNS group (90.05±2.32 s *vs*. 99.40±2.91 s, 122.20±2.96 s, *P*<0.05, *P*<0.01), while the scores of epileptic behaviour of the rats decreased in the tan-VNS group and the ta-VNS group (4.85±0.11 s *vs*. 4.20±0.17 s, 3.05±0.22 s, *P*<0.05, *P*<0.01). In comparison with the tan-VNS group, the latency of the first grand mal increased in the ta-VNS group (*P*<0.01), while the scores of epileptic seizure behaviour decreased in the ta-VNS group (*P*<0.01) (Figure 3).

### ta-VNS suppressed epileptiform activity in EEG tracings by increasing the firing rates of the NTS neurons

**Figure 4:**
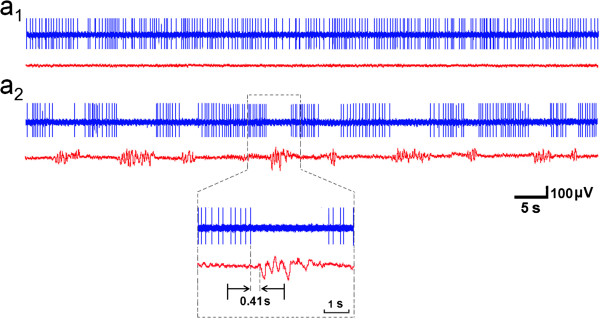
**Representative EEG tracing and firing of the NTS in one rat.** In **(a**_**1**_**)** and **(a**_**2**_**)**, the upper signal was the firings of the NTS neuron, the lower signal was EEG tracing. **(a**_**1**_**)** Before PTZ, **(a**_**2**_**)** After PTZ, Magnification: the decreasing of the firing rates of the NTS neuron was accompanied by an epileptic discharge in EEG tracing with 0.41 s delay.

**Figure 5:**
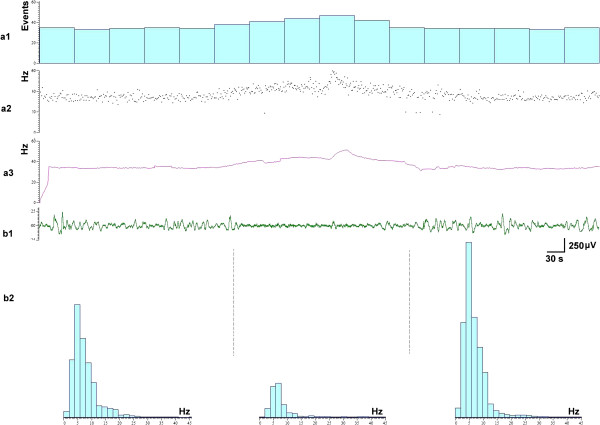
**Relationship between the firing of the NTS neurons and the epileptiform activity in EEG traces. (a**_**1-3**_**)** The discharges of the NTS neurons, **(a**_**1**_**)** Rates, **(a**_**2**_**)** Instantaneous frequency, **(a**_**3**_**)** Mean frequency. **(b**_**1**_**)** Epileptiform activity in EEG traces. **(b**_**2**_**)** Difference of spectral pattern of EEG when seizure off (left, right) or seizure on (middle).

Before intraperitoneal injection of PTZ, the firing rates of the NTS neurons and the EEG tracings were relatively stable (Figure 4a_1_). After intraperitoneal injection of PTZ, seizures manifested as highly synchronous, large-amplitude discharges in EEG tracings occurred. The average number of seizures per min was 6.12±0.57, and the average duration of one seizure was 4.07±0.34 s, which agrees with the results previously reported [13]. In comparison with background activity, the firing rates declined from 14.18±1.95 spikes/s to 8.18±1.95 spikes/s (*n*=31) after PTZ. Interestingly, when the firing rates of the NTS neurons decreased, the EEG epileptic activity increased with 0.37±0.12 s delay (Figure 4a_2,_ Figure 5a_1_). The changes of instantaneous frequency (Figure 5a_2_), mean frequency (Figure 5a_3_) of the NTS neurons were negative correlated with the amplitude of the epileptic EEG (Figure 5b_1_). The spectrum analysis of the EEG showed that when the epileptiform activity increased, 2–7 Hz EEG increased (Figure 5b_2_). The consecutive changes of the two signals in vivo suggested that the activity of NTS neurons was correlated with epileptiform activity.

**Figure 6:**
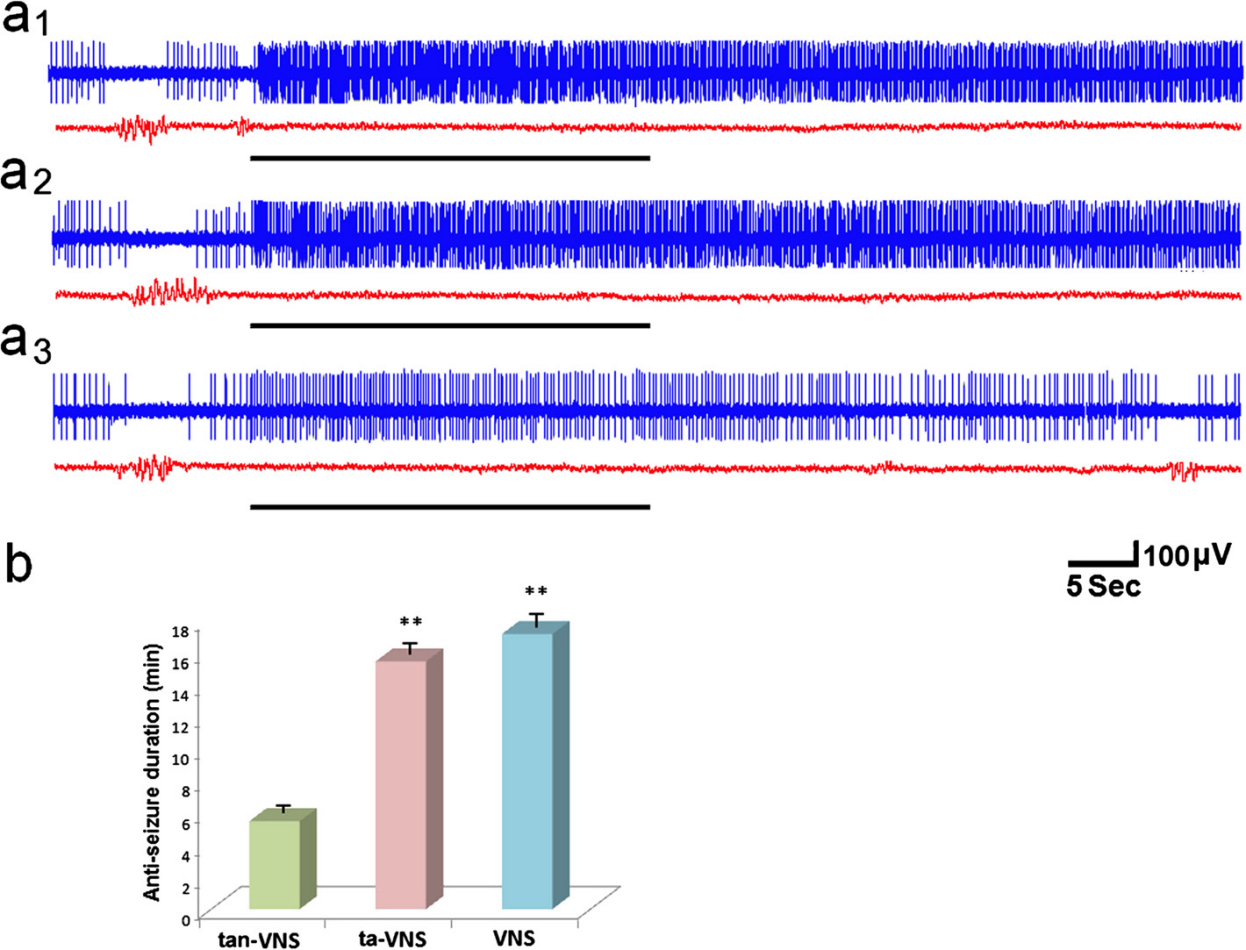
**VNS, ta-VNS, tan-VNS suppressed epileptiform activity by increasing the firing of the NTS. (a**_**1**_**)** VNS, **(a**_**2**_**)** ta-VNS, **(a**_**3**_**)** tan-VNS. Stimulations are indicated by horizontal bars. **(b)** Comparison of the anticonvulsant durations among three groups. **: *P* < 0.01, *vs*. tan-VNS, *n*=31 per group.

All three stimulations increased the firing rates of the NTS neurons and suppressed epileptiform activity in EEG traces. The firing rates of the NTS neurons increased 25 ±3% (*n*=17) by VNS, 22 ±2% (*n*=16) by ta-VNS, 12 ±1% (*n*=13) by tan-VNS. The anticonvulsant durations tan-VNS, ta-VNS and VNS were 5.46±0.49 min, 15.41±0.50 min, and 17.12±0.75 min respectively, which outlasted the periods during which stimulations were provided. The anticonvulsant durations of VNS and ta-VNS were significantly longer than that of tan-VNS (*P*<0.01). There was no significant difference between the anticonvulsant durations of VNS and ta-VNS (Figure 6).

### ta-VNS suppressed epileptiform activity in FPs of cortices

**Figure 7:**
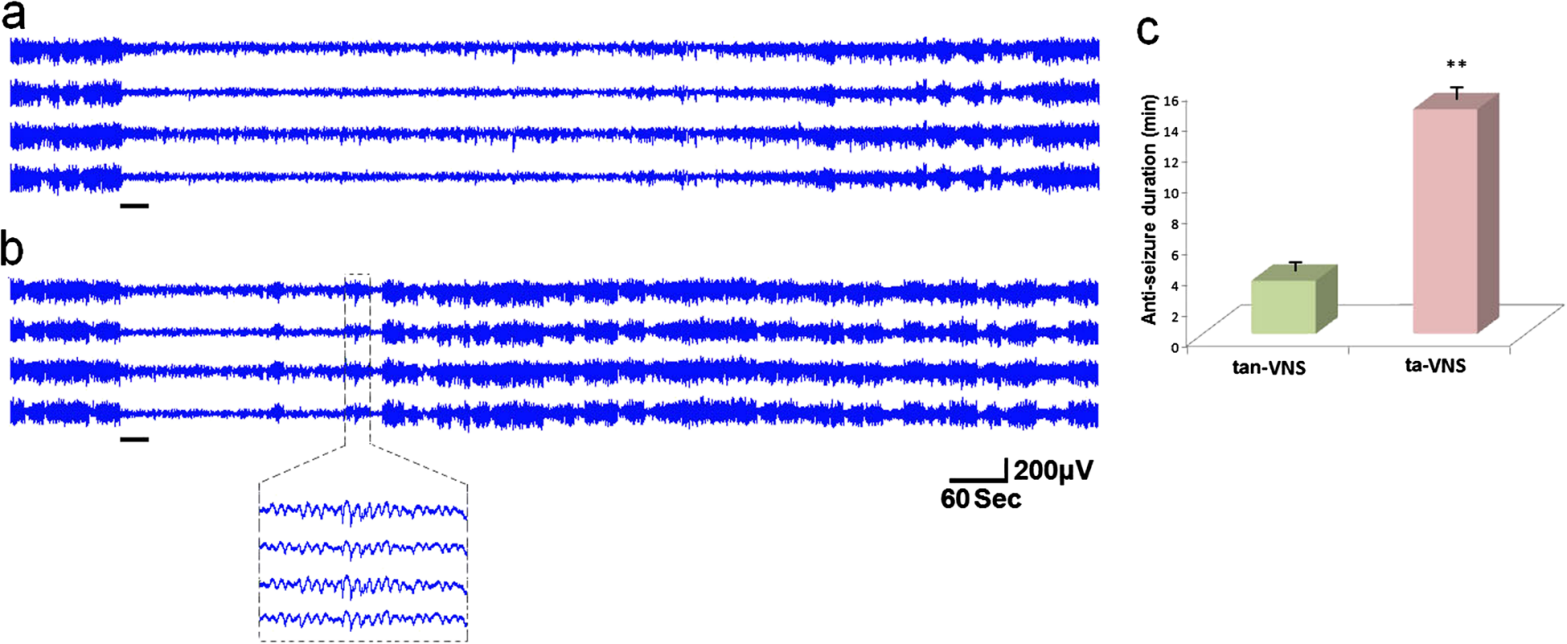
**Representative FP tracing of the cortex in one rat. (a)** ta-VNS. **(b)** tan-VNS. Stimulations are indicated by horizontal bars. **(c)** Comparison of the anticonvulsant duration between ta-VNS and tan-VNS. **: *P* < 0.01, *vs*. tan-VNS, *n*=12 per group.

After PTZ were administered with no stimulation provided, highly synchronous and large-amplitude epileptiform activity occurred in FPs tracings in somatosensory cortices. The rats were treated with ta-VNS or tan-VNS for 30 s. Compared with pre-stimulation, epileptiform activity in SI cortices were suppressed by ta-VNS or tan-VNS. The anticonvulsant duration of ta-VNS was much longer than that of tan-VNS (14.58±0.59 min *vs*. 3.45±0.34 min, *P*<0.01) (Figure 7).

### The anticonvulsant effect of ta-VNS was weakened by reversible cold block of the NTS

**Figure 8:**
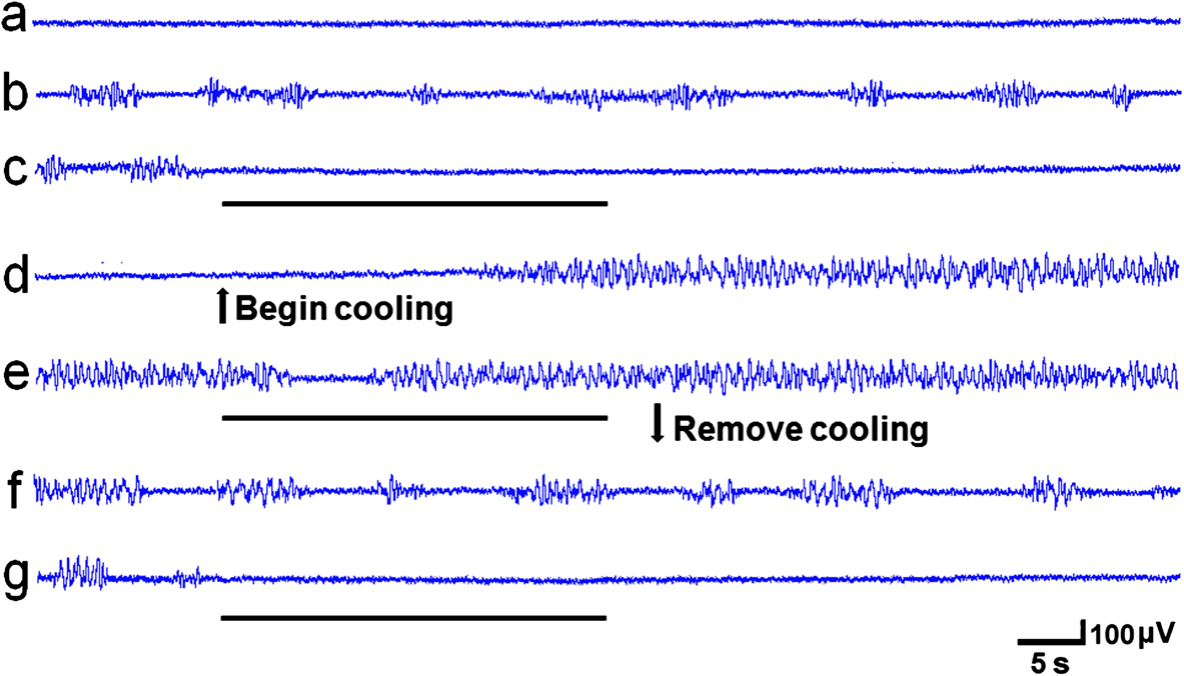
**Effect of reversible cooling the NTS neurons on the EEG changes in one rat. (a)** Before PTZ, **(b)** After PTZ, **(c)** ta-VNS before cooling the NTS, **(d)** Cool the NTS neurons without ta-VNS, **(e)** ta-VNS when cooling the NTS neurons, **(f)** Five min after removing the cooling, **(g)** ta-VNS after removing the cooling. VNS are indicated by horizontal bars.

Before PTZ, the EEG tracings were relatively stable. After PTZ, epileptiform activity occurred. Before cooling the NTS, ta-VNS suppress the epileptiform activity for 14.98±0.69 min. When cooling the NTS neurons, ta-VNS suppress epileptiform activity for 0.11±0.03 min, which is significant less than ta-VNS before cooling of the NTS neurons (*P*<0.01). After removing the cooling of the NTS, epileptiform activity recovered to the level before cooling without ta-VNS. And, ta-VNS suppressed epileptiform activity for 14.56±0.73 min, there is no significant difference in comparison with ta-VNS before cooling of the NTS neurons (*P*>0.05). In Figure 8 there is a procedure for one rat.

## Discussion

In the present study, we first observed the afferent nerve terminals in NTS projected from the ABVN. Further, it was also observed that ta-VNS suppressed epileptiform activity via activating firing of the NTS neurons. The anticonvulsant effect of ta-VNS was weakened by reversible cold block of the NTS.

As the only peripheral branch, the ABVN is the last phylogenetic remnant of the nerve which innervates the lateral line organs in fish and amphibia. It is a mixed nerve composed of the vagus nerve, the glossopharyngeal nerve and the facial nerve [5]. Anatomical studies showed that the ABVN was distributed to the posterior wall of the external auditory canal [7]. Recently, the relationship between the ABVN with the NTS was investigated. Brain stem potentials elicited by ta-VNS are considered to be conducted via the ABVN to the NTS [14-16]. Functional MRI studies also demonstrated that ta-VNS on the left tragus was associated with higher order relay nuclei of vagal afferent pathways [17]. We firstly observed the afferent projections from the ABVN to the NTS in the rats, which provides the anatomical evidence to support the AVAP.

VNS has been utilized in suppressing epilepsy for decades. The common parameters of VNS were 30 s on, and 5 min off. The pulse width of stimulation was 0.25-0.5 ms. The output currents were 0.25-4 mA in 0.25-mA steps [8]. As the primary vagal afferent nucleus, the NTS has been considered to play an important role in the mechanism of VNS for the treatment of epilepsy [10]. The NTS electrical stimulation interferes with epileptogenesis [18,19]. An increase in gamma aminobutyric acid transmission or a decrease in glutamate transmission in the medial subnulceus of the NTS reduces susceptibility to limbic motor seizures in the rats [20]. But the technique for VNS has some disadvantages such as surgery risks, high costs and unknown long-term effects of the electrical stimulation of the vagus on the central nervous such as sleep apnea, headache [21]. ta-VNS is performed on the peripheral branch of the vagus nerve and is easy to be removed, which may provide a less-invasive treatment for epilepsy by decreasing or eliminating these disadvantages.

In the present study, it was observed that ta-VNS pretreatment had anticonvulsant effect in awake animals, which was in accordance with previous VNS pretreatment study [22]. In our electrophysiological study, it was the first to reveal in vivo the relationship between the firing rates of the NTS neurons and the epileptiform activity in rats. The consecutive changes of the two signals suggested that the firing of NTS neurons was negatively correlated with epileptiform activity. We also found that ta-VNS substantially increase the firing rates of the NTS neurons, and suppresses epileptiform activity in in awake and anaesthetized rats, which supports the hypothesis that ta-VNS suppresses epileptiform activity via the AVAP.

Furthermore, prolonged anticonvulsant durations by VNS, ta-VNS and tan-VNS were observed in our study. tan-VNS was stimulated at the exterior margin of the auricle, which is mainly innervated by the great auricular nerve (GAN). Except for cervical vagus nerve, the ABVN, the central projections of the GAN in the solitary tract (SN) has also been shown [23]. Therefore, the prolonged suppression of epileptiform activity by these three stimulations may be correlated with prolonged increasing of firing rates of the NTS neurons.

There were limitations in the present study. Sodium pentobarbital was administered to prolong the anaesthetic state. It is of possibility that use of this agent altered the dynamics of PTZ-induced seizures [24]. In our experiment, after each administration of sodium pentobarbital, we began the stimulation only if the epileptiform discharges were stable, to avoid the effect of sodium pentobarbital on the anticonvulsant effect of ta-VNS. Though there was no statistical difference between the anticonvulsant duration of VNS and ta-VNS, we cannot make a conclusion that the anticonvulsant effect of ta-VNS is the same as that of VNS. Recently, it was found in a pilot study that an overall reduction of seizure frequency in five patients after 9 months of ta-VNS [25]. We have also reported the anticonvulsant effect of ta-VNS in pediatric epilepsy patients [26]. Further studies should be made on the long-term effect of ta-VNS, and to find optimized parameters of ta-VNS for seizure suppression.

## Conclusions

The relation between the auricular branch of the vagus nerve and the autonomic and central nervous system is herein referred to as the “auriculo-vagal afferent pathway” (AVAP). The present study firstly observed the afferent projections from the ABVN to the NTS in the rat, with provided the anatomical evidence to support the AVAP. ta-VNS can suppress epileptiform activity via the AVAP. Further studies should be performed on the long-term effect of ta-VNS, and find optimized parameters of ta-VNS for seizure suppression in clinic.

## Competing interests

The authors declare that they have no competing interest.

## Authors’ contributions

BZ designed the experiment; WH, XHJ, BZ, XLZ, LL, WZB, HB performed the experiments; WH, XHJ, BZ, XLZ performed the surgeries; WH, XHJ, WZB, BZ analyzed the data; All authors discussed the data and the analysis methods and contributed to the manuscript. All authors read and approved the final manuscript.
